# Correlation analysis of Serum NT-pro-BNP, IGFBP-7 and CTRP12 levels in chronic heart failure patients

**DOI:** 10.5937/jomb0-61393

**Published:** 2026-05-22

**Authors:** Zhen Fan, Heng Huang, Laoji Wang, Jierou Yan, Fei Zhao

**Affiliations:** 1 The Affiliated Chuzhou Hospital of Anhui Medical University, Department of Cardiology, Chuzhou City, China; 2 Western Theatre Command Air Force Hospital, Department of Laboratory Medicine, Chengdu City, China; 3 The First Affiliated Hospital of Anhui Medical University, Department of Cardiology, Hefei City, China; 4 The First People's Hospital of Xiaoshan District, Department of Cardiology, Hangzhou City, China

**Keywords:** C1q tumour necrosis factor-related protein 12, chronic heart failure, IGFBP-7, NT-pro-BNP, C1q protein povezan sa faktorom nekroze tumora 12, hronična srčana insuficijencija, IGFBP-7, NT-pro-BNP

## Abstract

**Background:**

To investigate serum N-terminal pro-brain natriuretic peptide (NT-pro-BNP), insulin-like growth factor binding protein-7 (IGFBP-7), and C1q tumour necrosis factor-related protein 12 (CTRP12) levels and significance in patients with chronic heart failure (CHF).

**Methods:**

The CHF group consisted of 116 CHF patients who received care at this hospital between October 2023 and March 2025. The patients were classified into Grade II (47 patients), Grade III (41 patients), or Grade IV (28 patients) based on the heart function classification of the New York Heart Association (NYHA). The control group consisted of 64 healthy patients who were physically examined in the hospital during the same period. Using multivariate logistic regression, the factors impacting MACEs in patients with congestive heart failure were examined. Through the use of the receiver operating characteristic (ROC) curve, researchers were able to assess the predictive power of blood NT-pro-BNP IGFBP-7, and CTRP12 for MACEs in chronic heart failure patients.

**Results:**

Comparing the CHF group to the control group, the LVEDD increased, the LVEF and serum CTRP12 decreased, and the levels of NT-pro-BNP, IGFBP-7, Hcy, and hs-CRP climbed (P&lt; 0.05). Among patients with CHF of different grades, serum NT-pro-BNP IGFBP-7, Hcy, hs-CRP and LVEDD levels were all lower in Grade II patients than in Grade III patients. Furthermore, the differences between any two grades were statistically significant (P&lt; 0.05). The levels of LVEF and serum CTRP12 in the MACE group decreased, the LVEDD increased, and the levels of serum Hcy, hs-CRP NT-pro-BNP and IGFBP-7 increased (P&lt; 0.05). MACEs in CHF patients were influenced by serum NT-pro-BNP IGFBP-7, and CTRP12 (P&lt; 0.05). The areas under the curve (AUCs) of serum NT-pro-BNP IGFBP-7, CTRP12 alone and the combination of the three for predicting MACEs in patients with CHF were 0.862 (95% CI: 0.786-0.919), 0.805 (95% CI: 0.721-0.872), and 0.860 (95% CI: 0.784-0.918) and 0.961 (95% CI: 0.908-0.988), respectively. The AUCs of the three combined predictions were significantly greater than those of the individual predictions of NT-pro-BNP IGFBP-7, and CTRP12 (Z = 3.050 , 3.883, 3.218, all P&lt; 0.05).

**Conclusions:**

Serum levels of IGFBP-7 and NT-pro-BNP increase in CHF patients, whereas the level of CTRP12 decreases. Additionally, once functional classification was applied, NT-pro-BNP IGFBP-7, and CTRP12 levels changed. The combined detection of these three parameters has better efficacy in predicting MACEs in patients with CHF.

## Abstract (sr)

**Uvod:**

Cilj je bio da se ispitaju nivoi i značaj serumskog N-terminalnog pro-peptida moždanog natriuretičkog hormona (NT-pro-BNP), proteina vezanog za insulinu sličan faktor rasta 7 (IGFBP-7) i C1q proteina povezanog sa faktorom nekroze tumora 12 (CTRP12) kod pacijenata sa hroničnom srčanom insuficijencijom (CHF).

**Metode:**

Grupa CHF obuhvatila je 116 pacijenata sa hroničnom srčanom insuficijencijom koji su lečeni u ovoj bolnici u periodu od oktobra 2023. do marta 2025. Pacijenti su klasifikovani kao "Grade" II (47 pacijenata), "Grade" III (41 pacijent), ili "Grade" IV (28 pacijenata) na osnovu klasifikacije srčane funkcije prema "New York Heart Association" (NYHA). Kontrolna grupa činilo je 64 zdrava pacijenta koji su u istom periodu obavljali preventivne preglede u bolnici. Korišćenjem multivarijatne logističke regresije analizirani su faktori koji utiču na pojavu glavnih kardiovaskularnih neželjenih događaja (MACE) kod pacijenata sa kongestivnom srčanom insuficijencijom. Kroz korišćenje ROC krive procenjena je prediktivna vrednost nivoa NT-pro-BNP IGFBP-7 i CTRP12 u serumu za MACE kod pacijenata sa CHF.

**Rezultati:**

U poređenju sa kontrolnom grupom, kod CHF grupe došlo je do povećanja LVEDD, smanjenja LVEF i serumskog CTRP12, dok su nivoi NT-pro-BNP IGFBP-7, Hcy, hs-CRP porasli (P&lt; 0,05). Kod pacijenata sa CHF različitih stepena, nivoi serumskog NT-pro-BNP IGFBP-7, Hcy, hs-CRP i LVEDD bili su niži kod pacijenata "Grade" II u odnosu na "Grade" III, a razlike između bilo koja dva stepena bile su statistički značajne (P&lt; 0,05). Nivoi LVEF i serumskog CTRP12 u MACE grupi su se smanjili, LVEDD je porastao, a nivoi serumskog Hcy, hs-CRP NT-pro-BNP i IGFBP-7 su se povećali (P&lt; 0,05). MACE kod pacijenata sa CHF bili su pod uticajem serumskih nivoa NT-pro-BNP IGFBP-7 i CTRP12 (P&lt; 0,05). Površine ispod krive (AUC) za predikciju MACE kod pacijenata sa CHF bile su: NT-pro-BNP - 0,862 (95% CI: 0,786-0,919), IGFBP-7 - 0,805 (95% CI: 0,721 -0,872), CTRP12 - 0,860 (95% CI: 0,784 -0,918), dok je kombinacija sva tri parametra dala AUC 0,961 (95% CI: 0,908-0,988). AUC kombinovane predikcije su bila značajno veća od pojedinačnih predikcija NT-pro-BNP IGFBP-7 i CTRP12 (Z = 3,050 ; 3,883; 3,218, svi P&lt;0,05).

**Zaključak:**

Serumski nivoi IGFBP-7 i NT-pro-BNP su povećani kod pacijenata sa CHF, dok je nivo CTRP12 smanjen. Takođe, nakon primene funkcionalne klasifikacije, nivoi NT-pro-BNP IGFBP-7 i CTRP12 su se menjali. Kombinovana detekcija ova tri parametra ima veću efikasnost u predviđanju MACE kod pacijenata sa CHF.

## Introduction

The advanced symptom of many cardiovascular disorders is chronic heart failure (CHF) [1]. Its main clinical features include symptoms such as shortness of breath, oedema, reduced exercise tolerance, and fatigue. If not treated in time, it can easily lead to adverse events such as cardiogenic lung disease, cardiogenic shock, and even sudden death [2]. Therefore, finding biological indicators that can accurately diagnose and assess patient prognosis is significant [3]. It is synthesised mainly by myocardial cells in the body and is decomposed under the action of proteolytic enzymes. Studies [4][5][6] have shown that NT-pro-BNP is involved in and affects the occurrence and progression of CHF. Insulin-like growth factor binding protein-7 (IGFBP-7) is a soluble secretory glycoprotein that is widely expressed in various tissues of the body and regulates a series of physiological processes, such as cell growth and adhesion, proliferation, apoptosis and angiogenesis. Studies [7][8][9] have shown that IGFBP-7 is involved in the occurrence of many cardiovascular diseases. C1q tumour necrosis factor-related protein 12 (CTRP12) is a member of the CTRP family and is highly homologous to adiponectin. It participates in and influences the occurrence and development of related diseases, such as diabetes, insulin resistance, and cardiovascular diseases [10][11]. Accurate assessment of the severity of the disease, early identification of high-risk patients and individualised management are the keys to improving patient prognosis. Currently, a traditional biomarker is crucial for the diagnosis and prognosis assessment of CHF [12]. However, its level is affected by multiple factors, and a single indicator has limitations [13]. Insulin-like growth factor binding protein 7 (IGFBP-7) is closely related to myocardial fibrosis and the inflammatory response and is considered a potential indicator of myocardial remodelling and cardiac dysfunction. C1q/tumour necrosis factor-related protein 12 (CTRP12), an adipokine with anti-inflammatory and metabolic regulatory effects, has increasingly drawn attention for its connection with cardiovascular protection and energy metabolism. However, studies [14][15][16] on the expression characteristics of serum IGFBP-7 and CTRP12 in patients with CHF and their intrinsic associations with NT-pro-BNP are still insufficient [17]. Therefore, this study aims to explore the correlations among NT-pro-BNF, IGFBP-7, and CTRP12 levels in the serum of patients and their association with disease severity. By jointly detecting these levels, the study seeks to reveal their synergistic mechanism and clinical value in the disease process. Our study provides a new scientific basis and diverse assessment perspectives for risk stratification, pathophysiological mechanism exploration and identification of potential therapeutic targets for CHF.

## Materials and methods

### Research subjects

One hundred sixteen CHF patients, with a body mass index (BMI) of 25.18±2.16 kg/m^2^, treated at our hospital between October 2023 and March 2025, were chosen as the CHF group. Sixty-two male patients and 54 female patients were included. Forty-eight patients had coronary heart disease, 36 had hypertension, and 31 had diabetes. All patients were classified into Grade II (47 patients), Grade III (41 patients), or Grade IV (28 patients). Another 64 healthy individuals, with a BMI of 25.16±2.14 kg/m^2^. The age ranged from 45 to 86 years, with an average of 66.63±18.59 years. There were 38 male patients and 26 female patients.

Inclusion criteria: (1) met the necessary diagnostic standards outlined in the »Guidelines for the Diagnosis and Treatment of Chronic Heart Failure« and had a history of heart disease symptoms; (2) Had symptoms such as edema, fatigue and shortness of breath lasting for more than half a year; (3) Had a significant decrease in exercise endurance; (4) had an ultrasonography electrocardiogram that showed heart failure symptoms, altered myocardial contractile and diastolic functions, and alterations in the left ventricle's structure and function.

Exclusion criteria: (1) Acute myocardial infarction or severe arrhythmia; (2) Severe liver or kidney dysfunction; (3) Percutaneous coronary intervention surgery one month before admission or acute myocardial infarction, unstable angina pectoris or cerebrovascular accidents within one month; (4) Accompanying haematological diseases; (5) Accompanying malignant tumours and other related diseases.

Our hospital's Medical Ethics Committee [HKYS-2025-A0224] gave its approval for this investigation. Every participant signed the informed consent form after their families explained the study to them.

### Echocardiography examination

The patients' end-diastolic diameter (LVEDD), left ventricular ejection fraction (LVEF), and those of healthy individuals were measured and recorded on the day of physical examination using the EPIQ 7C ultrasound diagnostic system (Philips, Netherlands).

### Serum-related index detection

On the second day of admission, four to six millilitres of fasting venous blood were drawn from CHF patients and from the control group in the early morning. The serum was obtained after centrifugation. Homocysteine (Hcy) and hypersensitive C-reactive protein (hs-CRP) levels were measured using a BS-600 M biochemical analyser (Shenzhen Mindray Biomedical Electronics Co., Ltd.), and the level of NT-pro-BNP was detected via a Cobas e601 electrochemiluminescence analyser (Roche, Switzerland). The levels of IGFBP-7 (a kit purchased from Shanghai Zhenke Biotechnology Co., Ltd.) and CTRP12 (a kit purchased from Xiamen Lun Changshuo Biotechnology Co., Ltd.) were determined via enzyme-linked immunosorbent assay. All laboratory indicators were tested by three skilled doctors from the hospital's laboratory department.

### Laboratory testing methods and reagents

### Testing methods

Enzyme-Linked Immunosorbent Assay was adopted. ELISA technology was used to detect the concentrations of N-terminal pro-B-type natriuretic peptide (NT-pro-BNP), insulin-like growth factor binding protein 7 (IGFBP-7), and C1q/ tumour necrosis factor-related protein 12 (CTRP12) in serum samples of patients with chronic heart failure. After adding the serum sample to be tested, the target antigen (NT-pro-BNP iGfBP-7 or CTRP12) is captured by the coated antibody. Wash the plate to remove unbound substances. Then add biotin-labelled specific detection antibodies to form a »capture antibody - antigen - detection antibody« complex. After washing the plate, add Streptavidin labelled with horseradish peroxidase (HRP) to bind with biotin. Rewash the plate to remove unbound substances. Finally, the tetramethyl-benzidine (TMB) substrate solution is added for the colour development reaction.

### Testing reagents and apparatus

1. NT-pro-BNP detection was conducted using the human NT-pro-BNP ELISA kit (Catalogue number: SEA479Hu) provided by Cloud-Clone Corp., Wuhan, China.
2. IGFBP-7 detection, the Elabscience® brand Human IGFBP-7 ELISA kit (Catalogue number: E-EL-H0010; Elabscience Biotechnology Inc., an American research and development company) is selected, located in Houston, TX, USA.
3. CTRP12 detection, the human CTRP12 ELISA kit (product number: CB-EL027015HU) produced by Wuhan Huamei Bioengineering Co., LTD. (CUSABIO).
4. The absorbance (OD value) of each well was determined at a wavelength of 450nm using the Multiskan FC microplate reader from Thermo Fisher Scientific (Waltham, MA, USA) (item No. 51119000CN).
5. Separation using an Eppendorf (Hamburg, Germany) Centrifuge (model Centrifuge 5810 R) at 3000 revolutions per separation center for 10 minutes.
6. Store samples using the Thermo Scientific™ Forma™ series -80°C ultra-low temperature refrigerator (Item No. 885003003).
7. The pipettes used are Gilson single-channel and multi-channel micropipettes (Gilson SAS from France).

### Follow-up investigation and analysis

After receiving standardised treatment and being released from the hospital, all CHF patients were monitored for 12 months. The patients were followed once every 3 months. Whether the patients developed MACE was recorded through phone calls, follow-up visits, etc. MACEs mainly include malignant arrhythmia, cardiogenic death and recurrent heart failure.

### Statistical methods

One-way analysis of variance was used for comparisons between several groups, independent sample t-tests for comparisons between two groups, and SNK-q tests for further pairwise comparisons. Multivariate logistic regression analysis was used to identify the factors that contribute to MACEs in patients with congestive heart failure. Serum NT-pro-BNP IGFBP-7, and CTRP12 were used to investigate the predictive power of MACE in CHF patients using the ROC curve.

## Results

### Comparison of cardiac function indicators and serum levels of NT-pro-BNP, IGFBP-7, CTRP12, Hcy and hs-CRP between CHF patients and the control group

Compared with those in the control group, the levels of LVEF and serum CTRP12 in the CHF group were lower, the LVEDD was greater, and the levels of serum NT-pro-BNP IGFBP-7, Hcy, and hs-CRP were greater. There was a statistically significant difference (P<0.05) (Table 1).

**Table 1:** Comparison of cardiac function indicators and serum levels of NT-pro-BNP IGFBP-7, CTRP12, Hcy and hs-CRP between CHF patients and the control group (x̄ ± s).

Group	n	LVEF<br>(%)	LVEDD<br>(mm)	Hcy<br>(μmol/L)	hs-CRP<br>(mg/L)	NT-pro-BNP<br>(pg/mL)	IGFBP-7<br>(ng/mL)	CTRP12<br>(ng/mL)
CHF<br>group	116	52.46±10.94	63.58±16.95	169.77±36.31	12.23±4.07	1587.40±294.68	70.80±19.54	2.12±0.78
Control<br>group	64	68.52±15.38	42.21±9.36	47.99±12.64	1.65±0.37	264.83±72.46	46.31±12.48	3.70±0.92
t		-8.127	9.325	25.950	20.723	35.281	9.053	-12.192
P		<0.001	0.001	<0.001	<0.001	<0.001	0.001	<0.001

### Comparison of serum cardiac function indicators and levels of serum NT-pro-BNP, IGFBP-7, CTRP12, Hcy and hs-CRP in CHF patients with different NYHA cardiac function grades

Among patients with CHF of different grades, the levels of serum NT-pro-BNP IGFBP-7, Hcy, hs-CRP and LVEDD were all Grade II patients < Grade III patients < Grade IV patients, and the levels of serum CTRP12 and LVEF were all Grade II patients > Grade III patients > Grade IV patients. Additionally, the differences between any two grades were statistically significant (P<0.05) (Table 2).

**Table 2:** Comparison of cardiac function indicators and serum levels of NT-pro-BNP IGFBP-7, CTRP12, Hcy and hs-CRP in CHF patients with different NYHA cardiac function grades (x̄ ± s).

NYHA Cardiac<br>function<br>classification	n	NT-pro-BNP<br>(pg/mL)	IGFBP-7<br>(ng/mL)	CTRP12<br>(ng/mL)	LVEF<br>(%)	LVEDD<br>(mm)	Hcy<br>(μmol/L)	hs-CRP<br>(mg/L)
Grade II	47	984.27±194.74	53.85±11.64	2.78±0.62	57.92±12.37	53.98±14.53	125.95±28.75	9.42±3.51
Grade III	41	1658.49±281.16	76.93±18.75	1.94±0.56	52.17±10.53	64.89±17.67	182.34±39.95	12.31±4.32
Grade IV	28	2495.70±430.65	90.26±23.61	1.28±0.45	43.72±9.16	77.77±19.96	224.92±43.67	16.82±4.64
F		230.267	40.997	65.783	14.572	17.212	67.014	28.669
P		<0.001	<0.001	<0.001	<0.001	<0.001	<0.001	0.001

### Comparison of general information, cardiac function indicators and levels of serum NT-pro-BNP, IGFBP-7, CTRP12, Hcy and hs-CRP between the MACE group and the non-MACE group

According to the occurrence of MACE within one year after discharge, Patients with CHF were split into two groups: 32 were MACE patients and 84 were non-MACE patients. Differences in age, sex, BMI, and combined coronary heart disease were not statistically significant, combined hypertension or combined diabetes between the MACE group and the non-MACE group (P>0.05). Compared with those in the nonMACE group, the levels of LVEF and serum CTRP12 in the MACE group decreased, the LVEDD increased, and there were significantly substantial (P<0.05) increases in serum Hcy, hs-CRP NT-pro-BNP and IGFBP-7 levels (Table 3).

**Table 3:** Comparison of general information, cardiac function indicators and serum levels of NT-pro-BNP IGFBP-7, CTRP12, Hcy and hs-CRP between the MACE group and the non-MACE group.

Group	n	Male/Female	Age<br>(years)	BMI<br>(kg/m^2^)	Combined with<br>coronary heart<br>disease	Combined with<br>hypertension	Combined<br>witt diabetes	Hcy<br>(μmol/L)
MACE<br>Group	32	18/14	64.58±18.74	25.95±6.18	18 (56.25)	10 (31.25)	10 (31.25)	228.64±49.50
Nonmace<br>group	84	44/40	62.91±18.20	24.89±6.37	30 (35.71)	26 (30.95)	21 (25.00)	147.34±31.29
x^2^/t		0.139	0.438	0.808	1.575	0.001	0.262	10.538
P		0.709	0.662	0.421	0.210	0.982	0.609	<0.001
Group	n	hs-CRP<br>(mg/L)	LVEF (%)	LVEDD<br>(mm)	NT-pro-BNP (pg/mL) IG		FBP-7<br>(ng/mL)	CTRP12<br>(ng/mL)
MACE<br>Group	32	17.36±5.40	44.71±9.52	68.94±18.43	1895. 18±320.62		78.49±23.35	1.87±0.64
Nonmace<br>group	84	10.27±3.56	55.41±11.84	61.54±15.14	1470. 15±266.35		67.87±18.42	2.21±0.85
x^2^/t		8.240	-4.576	2.212	7.252		2.571	-2.05
P		<0.001	0.001	0.029	<0.001		0.011	0.043

### Determinants of MACEs in CHF patients were examined using multivariate logistic regression

The collinearity test revealed that hs-CRP and Hcy had a collinear relationship with CTRP12 (tolerance =0.095, 0.075, and VIF=10.53, 13.33), and LVEF and LVEDD had a collinear relationship with NT-pro-BNP (tolerance =0.081, 0.064, and VIF = 12.3 5, 15). Thus, whether MACE occurred in CHF patients was the dependent variable for multivariate logistic regression analysis (occurrence =1, nonoccurrence =0), while the independent variables were solely NT-pro-BNP IGFBP-7, and CTRP12 (all inputs had original values). The findings showed that MACEs in CHF patients were influenced by serum NT-pro-BNP IGFBP-7, and CTRP12 levels (P<0.05) (Table 4).

**Table 4:** Multivariate logistic regression analysis of the influencing factors of MACE in patients with CHF

Factor	β	SE	Wald X2	P	OR	OR95%CI
NT-pro-BNP	0.848	0.307	7.630	0.006	2.335	1.279~4.262
IGFBP-7	0.953	0.365	6.820	0.009	2.594	1.268~5.305
CTRP12	-0.361	0.122	8.754	0.003	0.697	0.549~0.885

### Efficacy of serum NT-pro-BNP, IGFBP-7 and CTRP12 in predicting the occurrence of MACE in patients with CHF

The ROC curve was plotted according to whether MACE occurred in CHF patients (occurrence = 1, nonoccurrence =0) as the state variable and serum NT-pro-BNP IGFBP-7 and CTRP12 as the test variables. The areas under the curve (AUCs) of serum NT-pro-BNP IGFBP-7, CTRP12 alone and the combination of the three for predicting MACE in patients with CHF were 0.862 (95% CI: 0.786-0.919), 0.805 (95% CI: 0.721-0.872), 0.860 (95% CI: 0.784-0.918) and 0.961 (95% CI: 0.908-0.988), respectively. The AUCs of the three combined predictions were significantly greater than those of the individual predictions of NT-pro-BNP IGFBP-7, and CTRP12 (Z=3.050, 3.883, 3.218, all P<0.05) (Table 5).

**Table 5:** The efficacy of serum NT-pro-BNP IGFBP-7 and CTRP12 in predicting MACE in patients with CHF

Indicator	AUC	AUC95%CI	Optimal cutoff value	Sensitivity (%)	Specificity (%)	Youden Index	P
NT-pro-BNP	0.862	0.786~0.919	1693.98 pg/mL	75.00	84.52	0.595	<0.001
IGFBP-7	0.805	0.721~0.872	68.79 ng/mL	87.50	60.71	0.482	0.001
CTRP12	0.860	0.784~0.918	2.11 ng/mL	93.75	63.10	0.569	0.001
Three joint	0.961	0.908~0.988	-	90.62	91.67	0.823	<0.001

## Discussion

CHF is a type of myocardial injury caused by factors such as myocardial infarction. As the condition progresses, it may lead to ventricular pumping or overfilling, resulting in severe insufficiency of blood circulation in the arterial system [18][19][20][21]. Moreover, the aetiology of CHF is complex, the course of the disease is long, and the prognosis is poor. Patients need to be hospitalised frequently and are prone to MACEs [22]. Consequently, it is crucial to find biological markers that can reliably forecast a patient's prognosis.

NT-pro-BNP is the amino-terminal fragment cleaved during the secretion process of B-type brain natriuretic peptide (BNP) [23]. Cardiomyocytes secrete it, and it enters the bloodstream through cellular stretching. Its secretion and release are closely related to ventricular dilation. Relevant studies [24][25][26] have shown that when cardiac function is impaired, NT-pro-BNP is synthesised and secreted in large quantities, and its level increases rapidly. As cardiac function improves, the serum NT-pro-BNP level in CHF patients also gradually increases [27]. Moreover, ROC curve analysis revealed that the NT-pro-BNP level may have particular guiding significance for disease development and prognosis evaluation in CHF patients [28][29][30]. Igfbp-7 is extensively expressed in various tissues and organs, including peripheral nerves, the digestive tract, and breast tissue, where it participates in regulating processes such as cell growth, proliferation, and apoptosis [31]. Abnormal expression of IGFBP-7 can cause cell cycle arrest, tissue fibrosis, and promote the production of inflammatory cytokines. It is related to cardiac structure, diastolic function and the prognosis of CHF patients, and elevated levels of this protein increase the occurrence of MACEs [32]. In addition, studies have shown that higher concentrations of IGFBP7 may lead to premature ageing of the myocardium, thereby causing myocardial fibrosis.

Patients with heart failure have serum IGFBP-7 levels that are noticeably higher than those of healthy people. The serum IGFBP-7 level in patients with CHF is significantly elevated and related to cardiac function grade [33]. Additionally, the MACE group's blood IGFBP-7 level was noticeably higher than the non-MACE group's. The AUC of the serum IGFBP-7 level for predicting MACEs in patients with CHF was 0.805, indicating that the level of serum IGFBP-7 has good predictive value for MACEs in these patients [34]. CTRP12 is distributed mainly in the adipose tissue and kidneys, and multiple factors influence its level in the body. CTRP12 has multiple physiological functions in the body, such as exerting anti-inflammatory effects and regulating glucose metabolism [35]. Moreover, research has shown that CTRP12 can promote the process of vascular regeneration and alleviate the symptoms of myocardial fibrosis after myocardial infarction. Patients with severe CHD had lower serum CTRP12 levels than patients with moderate CHD, and as the severity of CHD increases, the amount steadily declines [36]. The serum CTRP12 level in patients with CHF decreased, and as the cardiac function grade increased, the CTRP12 level gradually decreased [37].

In addition, this study revealed that in patients with CHF, the LVEF was significantly decreased, the LVEDD was increased, and the levels of serum Hcy and hs-CRP were significantly elevated, which were related to the cardiac function grade. Further verifying that LVEF, LVEDD, and the levels of serum Hcy and hs-CRP are related to the occurrence of CHF. However, the collinearity test revealed that hs-CRP and Hcy had a collinear relationship with CTRP12. LVEF, LVEDD and NT-pro-BNP have a collinear relationship [38]. Therefore, hs-CRP Hcy, LVEF and LVEDD were not included in the multivariate logistic regression analysis or ROC curve analysis. Hs-CRP and hcy have respective AUCs of 0.650 and 0.808 for forecasting a bad outcome. Additionally, the combined prediction of serum NT-pro-BNP IGFBP-7, and CTRP12 for the occurrence of MACEs in congestive heart failure patients had an AUC of 0.961, which was significantly greater than the three individual AUCs, suggesting that the combination of the three factors has high efficacy in predicting the occurrence of MACEs in CHF patients.

## Conclusion

Serum levels of IGFBP-7 and NT-pro-BNP rise in CHF patients, whereas the level of serum CTRP12 decreases. Moreover, NT-pro-BNP IGFBP-7, and CTRP12 changed with functional classification. The combined detection of these three factors has greater efficacy in predicting MACEs in patients with CHF. This study will subsequently expand the sample size to further verify the predictive value of serum NT-pro-BNP, IGFBP-7, and CTRP12 for the prognosis of CHF patients and conduct an in-depth analysis of their mechanism of action.

## Dodatak

### Conflict of interest statement

All the authors declare that they have no conflict of interest in this work.
